# Novel Coronavirus Disease 2019 (COVID-19) and Cytokine Storms for More Effective Treatments from an Inflammatory Pathophysiology

**DOI:** 10.3390/jcm10040801

**Published:** 2021-02-17

**Authors:** Shumpei Yokota, Takako Miyamae, Yoshiyuki Kuroiwa, Kusuki Nishioka

**Affiliations:** 1 Department of Pediatrics, Yokohama City University, Yokohama 236-0004, Japan; ykuroiwa@med.teikyo-u.ac.jp; 2 Fuji-Toranomon Children’s Center, Gotemba 412-0045, Japan; 3 Japan Medical Research Foundation (JMRF), Tokyo 135-0063, Japan; kusuki-nishioka@bz04.plala.or.jp; 4 Japan College of Fibromyalgia Investigation (JCFI), Tokyo 160-0022, Japan; 5 Pediatric Rheumatology, Institute of Rheumatology, Tokyo Women’s Medical University, Tokyo 162-0054, Japan; tmiyamae@twmu.ac.jp; 6 Chairman of Stroke Center, Teikyo University School of Medicine Mizonokuchi Hospital, Kawasaki 192-0395, Japan; 7 Japan Society of Neurovegetative Research (JSNR), Tokyo 170-0002, Japan; 8 Global Health Innovation Policy Program (GHIPP), National Graduate Institute for Policy Studies (GRIPS), Tokyo 106-0032, Japan; 9 American College of Rheumatology (ACR), Atlanta, GA 30319, USA; 10 St. Marianna University, Kawasaki 216-8511, Japan

**Keywords:** novel coronavirus diseases (or COVID-19), cytokine storm, innate immunity, mitochondrial dysfunction

## Abstract

The Novel Coronavirus Disease 2019 (COVID-19) has swept the world and caused a global pandemic. SARS-CoV-2 seems to have originated from bats as their reservoir hosts over time. Similar to SARS-CoV, this new virus also exerts its action on the human angiotensin-converting enzyme 2. This action causes infections in cells and establishes an infectious disease, COVID-19. Against this viral invasion, the human body starts to activate the innate immune system in producing and releasing proinflammatory cytokines such as IL-6, IL-1β, IL-8, TNF-α, and other chemokines, such as G-CSF, IP10 and MCPl, which all develop and increase the inflammatory response. In cases of COVID-19, excessive inflammatory responses occur, and exaggerated proinflammatory cytokines and chemokines are detected in the serum, resulting in cytokine release syndrome or cytokine storm. This causes coagulation abnormalities, excessive oxidation developments, mitochondrial permeability transition, vital organ damage, immune system failure and eventually progresses to disseminated intravascular coagulation and multiple organ failure. Additionally, the excessive inflammatory responses also cause mitochondrial dysfunction due to progressive and persistent stress. This damages cells and mitochondria, leaving products containing mitochondrial DNA and cell debris involved in the excessive chronic inflammation as damage-associated molecular patterns. Thus, the respiratory infection progressively leads to disseminated intravascular coagulation from acute respiratory distress syndrome, including vascular endothelial cell damage and coagulation-fibrinolysis system disorders. This condition causes central nervous system disorders, renal failure, liver failure and, finally, multiple organ failure. Regarding treatment for COVID-19, the following are progressive and multiple steps for mitigating the excessive inflammatory response and subsequent cytokine storm in patients. First, administering of favipiravir to suppress SARS-CoV-2 and nafamostat to inhibit ACE2 function should be considered. Second, anti-rheumatic drugs (monoclonal antibodies), which act on the leading cytokines (IL-1β, IL-6) and/or cytokine receptors such as tocilizumab, should be administered as well. Finally, melatonin may also have supportive effects for cytokine release syndrome, resulting in mitochondrial function improvement. This paper will further explore these subjects with reports mostly from China and Europe.

## 1. Introduction

COVID-19, which is caused by an infection of novel coronavirus (SARS-CoV-2), began to spread from Wuhan City, Hubei Province in China in November 2019 and explosively spread around the world in a short period afterward. On 11 March 2020, the World Health Organization (WHO) declared that it was officially a pandemic [1].

The disease spread is similar to the outbreak of SARS in November 2002, which began in Guangdong Province, China, and expanded to 32 regions and countries, mainly in East Asia and Canada, as well as the MERS outbreak, which began in Saudi Arabia in September 2012. The three viruses have similar clinical symptoms and epidemiological characteristics [2,3,4]. Moreover, COVID-19 also seems to have a zoonotic source caused by a virus belonging to the β-coronavirus group (CoV). In severe cases, respiratory manifestations, such as acute respiratory distress syndrome (ARDS) and various central nervous system symptoms, are shared commonalities [5,6].

In clinical situations, the pathological processes of infectious diseases can have inflammatory stages present simultaneously. Pathological examination of the affected lungs among COVID-19 patients showed cellular fibro-mucinous secretions causing diffuse alveolar damage to both lungs, infiltration of inflammatory mononuclear cells, and detachment of type II alveolar epithelium and hyaline membranes. This closely resembles the pathology of ARDS and the lung pathology of SARS and MERS [7].

Increased levels of both proinflammatory cytokines (IL- 6, IL-1β, IL-8 and TNF-α) and chemokines (GCSF, IP10 and MCPl), were detected in the plasma of COVID-19 patients in intensive care units [4,8,9]. Thus, the pulmonary lesions seen in COVID-19 patients are presumed to be due to cytokine storms.

Cytokine release syndrome (CRS) often appears in paediatric rheumatic diseases, typically in systemic juvenile idiopathic arthritis (sjia) and childhood-onset systemic lupus erythematosus (c-SLE). This pathological condition is also known as macrophage activation syndrome (MAS) in the paediatric field [10]. Even in infectious diseases caused by Gram-negative bacteria, lipopolysaccharides (LPS), a component of Gram-negative bacteria’s outer membrane, induce cytokine storms during sepsis [11]. Hemophagocytic lymphohistiocytosis (HLH), which is also a pathological condition of CRS, stems from infectious diseases in primary immunodeficiency and from Epstein-Barr virus infections [12]. Although rheumatoid arthritis (RA) is a chronic inflammatory disease, the reasons behind its chronicity remain unknown. In the case of sjia, it is also a challenging matter as to why the inflammation suddenly converts into such a severe and lethal cytokine storm. Likewise, in SARS-CoV, MERS-CoV, and now with SARS-CoV-2 infections, the key factors that cause the switch from infectious states into cytokine storms are still unknown.

A possible reason is a breakdown of the innate immune system’s regulatory functions that produce and secrete various proinflammatory cytokines and chemokines. Thus, therapeutic strategies should urgently eliminate the overproduced proinflammatory cytokines and chemokines and suppress their further production [13]. On the other hand, it is possible to accurately determine the state of cytokine storms by monitoring several blood tests at the bedside. According to the precise evaluations of these parameters, improvements in CRS can be expected by introducing appropriate treatments at an early stage.

On that note, there have been recent reports that mitochondria are involved in the activation and regulation of the innate immune system, as well as the amplification of inflammation and its progression into chronicity [14]. Mitochondria are organelles that supply more than 95% of the body’s energy in the form of adenosine triphosphate (ATP) to humans. They also actively regulate the innate immune system against organ damage and release cell debris due to infectious and non-infectious diseases, resulting in the release of mitochondrial proteins (n-FP, Cardiolipin, etc.) and mitochondrial DNA (mtDNA). These processes contribute to damage-associated molecular patterns (DAMPs), which activate the innate immune system, including toll-like receptors (TLRs), NOD-like receptors (NLRPs) and the cyclic GMP-AMP synthase (cGAS) stimulator of interferon genes (STING) (cGAS-STING) pathway. This results in the production of proinflammatory cytokines, type I interferons and chemokines [15].

Various intrinsic and extrinsic stressors due to SARS-CoV-2 infections are also related to mitochondrial dysfunction and damage. When mitochondrial proteins and mtDNA are released, chronic inflammation occurs. With regards to acute inflammation, CRS seen in COVID-19 could be induced by pathological conditions.

In this paper, we will discuss the pathogenesis of cytokine storms associated with COVID-19, clarify how to evaluate the pathophysiology that changes from time to time at bedside, and discuss how to choose treatment methods aimed at saving lives from CRS/multiple organ failure (MOF) in serious cases of COVID-19.

## 2. Novel Coronavirus Disease and COVID-19

The outbreak of SARS-CoV-2 that began in Wuhan City, China, in November 2019 is spreading worldwide. This Novel Coronavirus Disease is also known as COVID-19. As of 12 October 2020, 37 million have been infected worldwide, and over 1 million have died.

So far, seven types of coronaviruses are known to be involved in human infectious diseases. Four of these are common cold viruses that infect the upper respiratory tract and account for 20–35% of the viruses which cause cold epidemics. On the other hand, the remaining three viruses—SARS-CoV, MERS-CoV and SARS-CoV-2—are known to cause lower respiratory tract infections, exhibit extremely severe ARDS and cause MOF [5,6].

SARS-CoV-2 is a virologically positive-strand RNA virus belonging to the β-coronavirus group, along with SARS-CoV and MERS-CoV. SARS-CoV-2 acts on the ACE2 receptor in humans, which is greatly involved in cardiac function and blood pressure adjustment. In COVID-19 cases, the spike protein (S protein) of the crown (corona)-like projection of the viral envelope binds to the receptor, and the RNA genome of the virus enters into the cell [16,17]. The affinity of the S protein of SARS-CoV-2 with the ACE2 receptor is reportedly very high [18]. ACE2 receptors are expressed in 83% of alveolar epithelial cells, meaning that the respiratory tract and alveolar epithelial cells are the primary targets of SARS-CoV-2 infection. At the same time, however, these receptors are also expressed on cell surfaces of the heart, kidney, vascular endothelium and gastrointestinal tract.

All coronaviruses are zoonotic infection viruses that appear to originate from bats, but infection shows strict specificity for certain animal species. When the virus mutates genetically and starts to infect animals of other species, it may exhibit severe pathogenicity at first, whereafter it then tends to become familiar with the other species. However, SARS-CoV-2 is still exhibiting this intense pathogenicity in humans worldwide, and time is of the essence to raise public awareness in the medical community to discuss and understand its pathophysiology to mount a proper response [19].

## 3. Pathological Changes

There have been several extensive pathological reports of COVID-19 initially from China [20,21]. In a histopathological report by Xu et al., both lungs had diffuse impairments in the alveolar space and wall. Infiltrates of cellular components with fibro-mucinous exudation were also seen. The right lung showed detachment of alveolar cells, had a vitreous formation and was diagnosed as ARDS. Meanwhile, the left lung had pulmonary edema, hyaline membrane formation, and was considered to be in the initial stage of ARDS.

Moreover, inflammatory mononuclear cells and lymphocytes infiltrated the interstitial tissue. Multinucleated syncytial cells were observed in abnormally enlarged respiratory tract spaces, and alveolar cells with enlarged nuclei were seen. There were also traces of viral infection in giant cells. These histological findings were similar to those of SARS-CoV and MERS-CoV. In liver tissue, moderately severe micro-vesicular deposits were observed, although changes in the follicles and portal veins were small. From these findings, SARS-CoV-2 infection or drug-induced liver damage was suspected. The cardiac tissue showed infiltration of inflammatory mononuclear cells in the interstitial tissue, but there was no damage in the heart muscle itself.

According to China’s “New Coronavirus Pneumonia Treatment Guidelines” [22], significant growth of type II alveolar epithelial cells is frequently observed in the lungs. Inclusion bodies are often observed in these type II alveolar epithelial cells and macrophages, while SARS-CoV-2 particles are sometimes observed in the cytoplasm with an electron microscope. Bleeding and necrosis occur in lung tissues, and haemorrhagic infarction is also seen. In the pancreas, atrophy infiltrated macrophages and phagocytosis were clearly observed. The number of lymphocytes in the pancreas, pulmonary lymph nodes and bone marrow decreased significantly. Both CD4^+^T and CD8^+^T cells were decreased in the pancreas and peripheral lymph nodes. In the bone marrow, pancytopenia was observed. Degeneration and necrosis of cardiomyocytes could be seen in the heart, while infiltration of a small number of monocytes, lymphocytes and neutrophils was seen in the interstitial tissue. Protein exudate appeared in the Bowman’s capsule of the kidneys, the tubular epithelium was denatured and desquamated, and hyaline casts were also seen. Hyperemia and edema were present in the brain tissue, while neuronal degeneration was also partially observed.

## 4. Pathophysiology of COVID-19

SARS-CoV-2 has the highest level of homology with SARS-CoV. Thus, it is important to take into account the Chinese social experiences and virological findings during the 2002 SARS-CoV epidemic, as well as their recent reports on COVID-19, since China experienced this earlier than other nations [1,2,9,13].

These severe coronavirus infectious diseases’ pathophysiology is an intense inflammatory condition caused by the infection and rapid growth of the virus in vivo. ARDS occurs as the primary manifestation of multiple organ disorders that follow CRS. If the reaction becomes excessive and out of control, it could eventually lead to MODS and MOF, resulting in death. According to Yang’s report [23], SARS-CoV-2 infects the respiratory tract, alveolar epithelial cells and vascular endothelial cells due to early rapid viral replication, which then induces severe apoptosis (programmed cell death) and vascular leakage.

Moreover, the infection of macrophages with SARS-CoV-2 promotes the activation of NLRP3 inflammasome and causes pyroptosis and the production and secretion of IL-1β. Pyroptosis is a programmed cell death wherein macrophages which induce inflammation by IL-1β/IL-18 secretion inhibit the viruses’ growth [24].

According to Huang et al. [8], clinical symptoms begin with a high fever, cough, severe muscle pain, dullness and lassitude. Then, respiratory disturbances appear, and chest CT findings reveal pneumonia. During this period, significant decreases in the number of white blood cells and lymphocytes, elongated PPT, increased D-dimer titer, decreased platelet count, increased alanine transaminase (ALT) titer, gradual increases of (aspartate transaminase (AST)/lactate dehydrogenase (LDH)) titer, and decreased albumin concentration become apparent. Plasma levels of IL-1β, IL-lRa, IFN-γ, IP10, MCPl, IL-8, IL-10, TNF-α, etc., also increase. The levels of these inflammatory cytokines and chemokines increased prominently in intensive care unit (ICU) patients (who all had ARDS) were compared with non-ICU patients.

Remarkable viremia was demonstrated by the observable increase of viral RNA in the blood. Myocardial injuries and secondary infections in these patients followed afterward. These results show that SARS-CoV-2 infection can induce remarkable cytokine storms through the overactivation of innate immunity. On the other hand, dysfunction of the adaptive immune system was presumed if significant decreases in the number of lymphocytes were observed.

Zhang et al. [25] considered IL-6 as the central cytokine involved in the excessive inflammatory pathology of the cytokine storms seen in severe cases of COVID-19. In the “New Coronavirus Pneumonia Treatment Guidelines” [22], tocilizumab, an IL-6 receptor antagonist, was used in 21 severe ICU patients. When 19 of these cases were out of danger, IL-6 was identified as a leading cytokine for patients in advanced stages of the disease. However, the clinical efficacy of tocilizumab in the treatment of COVID-19 are limited at that time.

## 5. Innate Immunity and Inflammatory Cytokines

**Figure 1:**
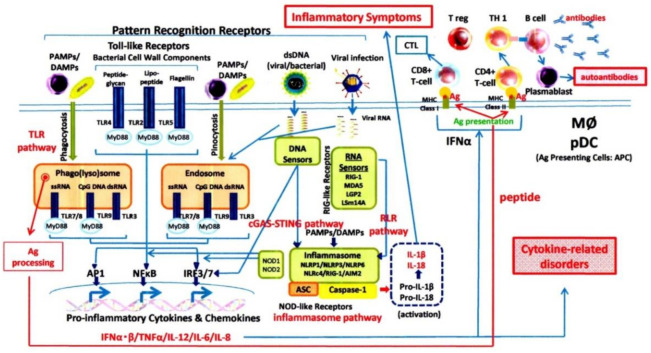
The innate immune system and acquired immune system.

The human body responds to stressors, such as environmental conditions, by activating the immune system to maintain homeostasis despite the stress. Such stress can be caused by bacterial and viral infections, the external environment (exogenous), and the body’s internal environment (endogenous) (Figure 1). Immune responses are divided into two major stages: innate immunity and adaptive immunity [26]. Innate immunity causes inflammation, leading to biological defenses, while adaptive immunity processes antigens such as bacterial components and viral capsids/envelopes to perform antigen-specific immune responses.

Pattern recognition receptors (PRRs) [27], such as toll-like receptors and NOD-like receptors, sense stress molecules in innate immunity. They process the preserved molecules of pathogens (LPS of Gram-negative bacteria, peptidoglycans of Gram-positive bacteria, etc.), detect these as “foreign bodies” (pathogen-associated molecular patterns, also known as PAMPs), then initiate inflammation. For endogenous stress, damage-associated molecular pattern molecules, which are composed of released damaged-cell components and other cell debris released under the conditions of cell stress, are recognised and then, in turn, mediate inflammatory responses. Ordinarily, the innate immune responses, being initiated by both the recognition of PAMPs and DMAPs along with the recruitment of inflammatory cells, are carried out very quickly. Afterward, there is a production and release of proinflammatory cytokines and chemokines, which mounts the overall inflammatory response. At the same time, antigen processing is carried out by both phagocytic and dendritic cells. The processed information then provides antigen-specific adaptive immunity accompanied by active type I interferon (IFN–α). Furthermore, two new functions of the mitochondria have recently been discovered: stimulatory functions of the innate immunity are associated with mitochondrial dysfunction. In contrast, regulatory functions of the innate immunity are associated with normal mitochondrial functions [28].

### Pattern Recognition Receptors (PRRs) and Innate Immunity

(1) Toll-like receptors (TLRs)

The inflammatory process begins when DAMPs and PAMPs bind to PRRs. TLRs are one of the PRRs and thus far, 10 functional TLRs have been identified in humans. TLR l, 2, 4, 5 and 6 are expressed on the cell’s surface, while TLR 3, 7, 8 and 9 are found in endosomes. When TLRs are activated, NFκB is induced in the nucleus via the signal transduction system, thus prompting the transcription of cytokine genes. Each TLR has a distinct function in terms of PAMP recognition and immune responses: TLR3 recognises dsRNA, while TLR4 has lipopolysaccharide (LPS) acting as a ligand. Mitochondria are also involved in the activation of TLR9 and TLR4 [28,29].

TLR7 is activated by viral nucleic acid, and the involvement of mitochondrial membranes in modulating TLR signalling has been reported. Also, DAMPs and PAMPs are involved in producing cytokines by activating transcription factor NFκB, similar to endosomes when they are digested in phagocytic cells such as macrophages. Simultaneously, the peptides that are processed in phagocytic cells lead to the activation of the acquired immune system, which recognises specific antigens.

(2) NLRP3 inflammasome

The NLRP3 inflammasome was initially considered a molecular complex related to the autoinflammatory disease cryopyrin-associated periodic syndrome (CAPS) caused by a genetic mutation. However, in recent years, it has come to be considered a molecular complex involved in infectious diseases and various chronic diseases with inflammatory pathologies, such as metabolic diseases, ischaemia-reperfusion injury, arteriosclerosis and Alzheimer’s disease [30].

The activation of NLRP3 occurs through two steps; the priming step and the activation step. In the priming step, NLRP3 is activated by TLR activation and the binding of cytokines to membrane receptors. Then, NLRP3 activation occurring as viral RNA is injected into cells and DNA is derived from bacteria. During the activation phase, a functional inflammasome following the formation of the ((NLRP3)—(cardiolicin)—(pro-caspase-1)—(ASC)) complex converts inactive pro-caspase-1 to its active form. Then, the activated caspase-1 enzymatically cleaves pro-IL-lβ and pro-IL-18, transforming these precursor forms into functional IL-lβ and lL-18, respectively, after which they are secreted extracellularly.

NLRP3 is also present in mitochondria, and together with the production and secretion of IL-lβ and IL-18, the cells are brought to pyroptosis, a form of programmed cell death. This process prevents the further intracellular proliferation of viruses. Furthermore, mitochondrial fracturing products and mitochondrial DNA contribute even more to the activation of innate immunity [24].

(3) Retinoic acid-inducible gene-like receptors (RLRs)

Since the genomes of viruses repeatedly propagate in cells, cytokine induction by TLRs expressed on the cell surface is impossible. However, there is a system, separate from TLRs and inflammasomes, that recognises the viral genome’s entry into the cell. This system is able to produce and secrete type I interferons (IFNs) [31]. For the genome of RNA viruses, RLRs (RIG-I, MDA5, LPG2, etc.) act as sensors for viral RNA. The system activates transcription factors IRF3, IRF7 and NFκB via the signalling pathway and also performs the transcription of type I IFNs and other proinflammatory cytokines.

(4) cGAS-STING pathway

This system recognises viral DNA in cells and is involved in the production and release of type I IFNs [32]. This system is also activated when impaired or dysfunctional mitochondrial DNA (mtDNA) is released into the cytosol. Viral DNA and mtDNA in cells bind to cGMP synthase (cGAS) and activate them, which, in turn, activate STING by turning GTP and ATP into cGAMP (cGMP-AMP). In this pathway, transcription factors such as IRF3 and NFκB are activated, resulting in the upregulation of interferons and other inflammatory genes.

## 6. Mitochondria and Innate Immunity

**Figure 2:**
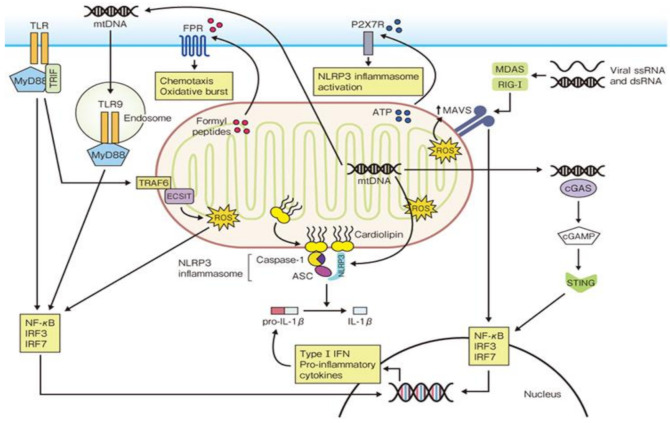
Mitochondrial regulation of innate immune responses.

Mitochondria are subcellular organelles that supply energy in the form of ATP to all of the eukaryotic organelles when energy needs have to be met. For ATP production, the matrix must be in a negatively-charged state because ATP synthesis involves the transfer of electrons in electron transmission chains (Figure 2). This negatively-charged state can collapse due to various stress factors such as antioxidants, oxidative phosphorylation substrates and membrane-unbound substances [33]. When this happens, ATP production disorder occurs, causing extensive damage to the human body because of reactive oxygen species’ production and secretion (ROS). In addition, impaired mitochondria lose their membrane function. Consequently, their components such as cardiolipin, ROS and mtDNA escape into the cytosol and are released into the bloodstream, resulting in the activation of the innate immune response as DAMPs [28].

Cardiolipin is ordinarily detected in animals’ cardiac muscle tissue, but it is also present in the mitochondrial inner membrane. Mitochondria dramatically perform a structural transformation (fission-and-fusion) to fulfil this function because of mitochondrial cardiolipin’s special functions. In response to mitochondrial dysfunction, cardiolipin moves to the outer mitochondrial membrane and ties the NLRP3 inflammasome to the mitochondrion, triggering its activation.

Mitochondria are derived from proteobacteria that entered intracellular symbiosis with eukaryotes around 2 billion years ago. It has its own DNA (mtDNA), which divides and proliferates, but because this DNA is bacteria-derived, it cannot be cleaved by human nucleases. When it encounters cell death and mitochondrial dysfunction, mtDNA escapes into the cytosol. Upon entering the systemic circulation, it strongly activates the innate immune response through the NLRP3 inflammasome, TLR9/TLR4, RLR, etc. There are reports of increased mtDNA in plasma obtained from patients with external injuries, RA, femoral fractures, shock and other conditions in clinical situations.

Mitochondria are intracellular organelles present in individual cells throughout the human body. Thus, when various cellular disorders and cell death occur, especially during a cytokine storm, mitochondrial-derived calmodulin and mtDNA are presumed to have extremely serious and exaggerated roles in the vicious cycle of excessive production of proinflammatory cytokines, IFNs and chemokines.

## 7. Cytokine Storm and Its Pathophysiology from the Viewpoint of Physical Reactions

When regulation of the innate immune responses spirals out of control and fails, the exaggerated production and secretion of proinflammatory cytokines and chemokines cause progression into CRS. Then, the following processes occur: (1) DAMPs are released from the damaged cells and tissues; (2) Many PAMPs are released from infected tissues and microbial flora in the body; (3) Epithelial cell damage, endothelial cell destruction and mitochondrial dysfunction occur due to excessive cytokines and chemokines, so the innate and acquired immune functions fail.

However, CRS is not a disease; it is a pathophysiological condition that deteriorates from time to time. As a result: (1) Destroyed tissues and cells cause abrupt and massive increases of a wide range of cytopathic products AST/LDH and muscle-specific enzymes (creatine kinase (CK)/aldolase) due to apoptosis/necrosis/pyroptosis, etc.; (2) Widespread damage of endothelial cells causes the collapse of the coagulation-fibrinolysis system and the leakage of blood plasma out of the blood vessels, progressing into disseminated intravascular coagulation disseminated intravascular coagulation (DIC); (3) The innate immune responses are further exacerbated by mitochondrial dysfunction. Cardiolipin and mtDNA, which escape into the cytosol and plasma due to mitochondrial stress, cellular damage and cell death, further advance inflammatory pathophysiological situations.

Clinical symptoms such as fever, lassitude, fatigue, feelings of malaise, anorexia and increased levels of C-reactive protein (CRP) in inflammatory conditions are responsible for the high levels of plasma IL-6 and IL-1β. However, it is also possible to understand CRS’s degree and progression at the bedside by comprehensively considering changes in blood test values.

The rise of IFN-γ promotes activation of the vascular endothelial cells, which then induces HLA class I molecules and adhesion molecules’ expression. Activated endothelial cells found in an IFN-γ excess state then progress to collapse. Next, collagen fibres under the endothelial layer become exposed and bind with von Willebrand factor. At the same time, platelets adhere and produce clots. The exposed collagen then activates the intrinsic coagulation system, and together with tissue factors from the endothelial cells of the collapsed vasculature, the extrinsic coagulation system is activated. Finally, platelets and fibrin cover the cast-off regions of the endothelial cells.

Fibrin formation activates plasmin, which results in increases of FDP and D-dimer in the blood. When this coagulation fibrinolytic system collapses, microvascular coagulation disorder and DIC worsens in blood vessels. Also, excess IFN-γ activates macrophages in the bone marrow and promotes phagocytosis. This decreases the number of leukocytes and platelets in peripheral blood, causing the development of anaemia. When TNF-α binds to the receptors, the intracellular transmission system triggers the mitochondrial permeability transition, resulting in cytochrome C release. This then activates caspase-3 in the cells and induces apoptosis. As a result, AST/LDH titers increase rapidly and progressively. Similarly, when cell destruction in various organs progresses, CK/aldolase titers increase in muscle cell destruction, as does an increase of ALT in hepatocyte destruction. TNF-α induces ferritin from the reticuloendothelial system. Thus, ferritin levels are a good indicator of excessive TNF-α levels at the bedside. Another function of TNFα is to promote the elevation of triglycerides and decrease of cholesterol by inhibiting lipoprotein lipase activity in adipose tissue, meaning that these are also good indicators of cytokine storm pathology at the bedside. Also, there is a significant decrease in lymphocytes in COVID-19, and sufficient consideration is needed for secondary infections associated with acquired immune dysfunction.

SARS-CoV-2 infection exposes a large amount of PAMPs to the innate immune system, and the additional tissue and cellular disorder augments many DAMPs [34]. This results in the excessive secretion of proinflammatory cytokines and chemokines. Therefore, the apoptosis of cells and pyroptosis of macrophages end up inducing excessive inflammatory responses with the production of IL-1β/IL-18. Dysfunction progresses under the following conditions: cytokine storm state; ARDS from respiratory and alveolar epithelial cell damage; microvascular coagulation-fibrinolysis disorders from endothelial cell damage; mitochondrial mitophagy and catabolism. These are all part of a vicious cycle that eventually leads to MODS. Also, patients are more susceptible to secondary infections from the decrease in the immune system’s lymphocytes and dysfunction. Therefore, the exaggerated activation of innate immune responses during a SARS-CoV-2 infection leads to CRS, and thus, the progression from MODS to MOF over time can be more easily understood [35,36].

## 8. Treatments of Cytokine Storms in COVlD-19

The treatment strategies for COVID-19 can be divided into at least 3 categories: (1) Treatment of pathogens with antiviral drugs for SARS-CoV-2; (2) Cessation of cytokine storms; (3) Treatment of injured organs, such as respiratory system dysfunction due to ARDS, central nervous system dysfunction due to brain edema and coagulation-fibrinolysis system dysfunction due to DIC. Regarding the first strategy, various antiviral drugs are currently candidates, and clinical trials of favipiravir (Avigan^®^) developed by FUJIFILM Toyama Chemical Co., Ltd., Toyama, Japan, have begun [37]. Another strategy to prevent the binding of COVID-19 to ACE2 receptors could be using nafamostat (Futhan^®^) [38]. Regarding the third strategy, this falls under the responsibility of ICU management, and external polar membrane oxygenation (ECMO) is currently employed.

The following treatment strategies will focus on the second strategy, which concerns the cessation of cytokine storms and/or blocking the vicious cycle of exaggerated innate immune responses (Table 1).

### 8.1. Anti-IL-6 Therapy

#### 8.1.1. Tocilizumab (Actemra^®^)

Tocilizumab is a humanised anti-human IL-6 receptor monoclonal antibody and an anti-rheumatic drug approved by the Japanese Government for rheumatic diseases such as RA, JIA and Takayasu arteritis [55]. Systemic JIA (sJIA) is one of the three subtypes of JIA, and adult Still’s disease is thought to be an adult form of sJIA. During their clinical courses, these diseases can possibly undergo an acute conversion to MAS, which is one of the types of CRS.

Coronaviruses, such as SARS-CoV, MERS-CoV and SARS-CoV-2, induce prominent and frequently unmodifiable cytokinemia and chemokinemia due to excessive innate immune responses. A patient’s clinical course can take a sudden turn for the worse from CRS to ARDS and MODS/MOF. Eventually, these induce a series of conditions that can lead to death. As discussed earlier, this condition can result in the destruction of vascular endothelial cells, extravasation of plasma, organ tissue destruction, coagulation-fibrinolysis system failure, and a series of mechanical and functional disorders such as DIC—all of which can lead to collapse of the human body.

One of the leading cytokines in this cytokine storm condition is IL-6. Since the immune complexes of IL-6/IL-6 receptors need to bind to another cell-bound receptor, gp130, to transduce IL-6 messages, one of the emerging treatment strategies to suppress excessive IL-6 function is by blocking IL-6 receptors with monoclonal antibodies such as tocilizumab.

In a report [39] from China, 21 COVID-19 patients in ICUs with severe respiratory failure (and were not responsive to lopinavir and methylprednisolone) were treated tocilizumab. Clinical symptoms, oxygen saturation, laboratory findings and chest CT findings all improved the following day, and 19 patients (90.5%), including the 2 most severe cases, were discharged from the hospital within 13.5 days. The remaining two cases also improved after receiving more treatment in the hospital. In all cases, there were no findings that could be attributed to the side effects of tocilizumab. Tocilizumab is currently recommended as a therapeutic agent for severe COVID-19 in China and Italy [25,40,41]. However, a randomised, double-blind, placebo-controlled trial by the BACC Bay Tocilizumab Trial Investigators in the United States involving hospitalised COVID-19 patients in hyperinflammatory states failed to demonstrate a significant effect in preventing intubation or death in moderately ill hospitalised patients [42]. In EMPACTA (Evaluating Minority Patients with Actemra), a global, phase 3 clinical trial in hospitalised patients with COVID-19 pneumonia who were not receiving mechanical ventilation showed that the likelihood of progression to mechanical ventilation or death by day 28 was significantly lower among patients who received tocilizumab in addition to standard care, compared to those who received placebo plus standard care. Still, it did not improve survival [43]. On the other hand, in the most recent reports in January 2021, a REMAP-CAP trial that included less severely ill patients showed reduced mortality (from 35.8% to 27.3%) than standard care. Thus, tocilizumab has been recommended by the UK for COVID-19 [44].

#### 8.1.2. Sarilumab (Kevzara^®^)

Sarilumab is a fully human monoclonal antibody against IL-6 receptors, developed to treat rheumatoid arthritis [45]. It has reportedly shown a higher affinity for binding with IL-6R binding which translated into higher receptor occupancy and greater reduction in CRP levels compared to tocilizumab [46]. Along with tocilizumab, sarilumab was also studied in the previously mentioned REMAP-CAP trial, and reduced the time that critically-ill patients spent in intensive care by about a week [44].

### 8.2. Anti-IL-1 Therapy

Another central cytokine involved in cytokine storms is IL-1β. SARS-CoV and MERS-CoV are known to induce pyroptosis, which occurs with the release of IL-1β and IL-18. Thus, while emitting IL-1β, the infected macrophages biophylactically inhibit the intracellular growth of viruses. IL-1 stimulates endothelial cell-leukocyte adhesion and thromboxane B2 production by having a powerful action on vascular inflammation and platelet aggregation by mediating thrombi formation. The thrombogenesis observed in COVID-19 includes platelet and cell aggregation with clotting abnormalities [56].

#### 8.2.1. Anakinra (Kineret^®^)

Anakinra is a recombinant protein of IL-1 receptor antagonists (IL-lRa). It binds to IL-1 receptors with the same level of affinity as IL-lα and IL-1β. Anakinra is not yet approved for any diseases in Japan, but the U.S. FDA has approved it for RA and autoinflammatory diseases such as Muckle-Wells syndrome, familial cold autoinflammatory syndrome and neonatal onset multisystem inflammatory disease.

A phase III study has shown the effectiveness of anakinra for severe sepsis patients with cytokine storms. There was a significant improvement in the survival rates for MAS [57]. A French study reported that patients with COVID-19—associated pneumonia presenting with acute severe respiratory failure and systemic inflammation who received intravenous anakinra improved clinically (*p* < 0.01), with no deaths, significant decreases in oxygen requirements (*p* < 0.05), and more days without invasive mechanical ventilation (*p* < 0.06), compared with the control group [47].

#### 8.2.2. Canakinumab (Ilaris^®^)

Canakinumab is a human monoclonal antibody targeted at IL-1β approved for the treatment of autoinflammatory disorders such as CAPS, tumour necrosis factor receptor associated periodic syndrome (TRAPS), mevalonate kinase deficiency (MKD) and familial Mediterranean fever (FMF), as well as sJIA. It comes in a subcutaneous injection formulation and characterised by a long half-life of 26 days.

An observational, cohort, prospective study with 30 days of observation was conducted in patients hospitalised for COVID-19 pneumonia and treated with a single canakinumab dose. Although 13.6% of the patients died, 61.4% of cases experienced improved oxygen support [48]. Confirmation of the efficacy of canakinumab for COVID-19 requires further studies in randomised controlled trials.

### 8.3. Anti-TNF Therapy

TNF-α is a cytokine that can comprehensively induce other inflammatory cytokines, active pathologic factors in acute and chronic systemic inflammatory responses. In animal experiments of LPS-induced sepsis, TNF-α induces apoptosis in cells of various organs and plays a major role in the inflammatory responses of autoimmune diseases such as RA. However, very few studies have examined anti-TNF therapy as a potential treatment for COVID-19 thus far.

#### 8.3.1. Adalimumab (Humira^®^)

Adalimumab is a humanised anti-TNF-α monoclonal antibody used to treat autoimmune diseases such as RA, polyarticular JIA, Behçet’s disease, psoriasis, Crohn’s disease and ulcerative colitis. In SARS disease, serum levels of TNF-α increase to a moderate degree, but in COVID-19, TNF-α is increased to extremely high levels, correlating with disease activity.

There are a few case reports on using adalimumab in the acute setting in patients with COVID-19 [49]. In China, clinical trials of adalimumab for severe COVID-19 patients have already begun [50]. Moreover, Adalimumab for Coronavirus in Community Care (AVID-CC), one of the recent trials in COVID-19 patients, is currently in the process of evaluating the drug’s effect against respiratory failure in the community.

#### 8.3.2. Infliximab (Remicade^®^)

Infliximab is a chimeric monoclonal antibody indicated for inflammatory conditions, including RA and inflammatory bowel disease. In UK, the CATALYST randomised trial is currently investigating the use of infliximab in managing the inflammation of patients hospitalised with clinical features of COVID-19.

### 8.4. Janus kinase (JAK) Inhibitor: Baricitinib (Olumiant^®^)

When inflammatory cytokines such as IL-6 and TNF-α induce inflammatory responses, they bind to the cell surface receptors and activate intracellular signalling systems. Intracellular tyrosine kinases such as Janus kinase (JAK), bind to the intracellular portion of these receptors.

When these cytokines bind to each receptor, phosphorylation of transcription factor signal transducers and activators of transcription (STAT) is induced along with JAK phosphorylation. Then, the phosphorylated STAT forms a dimer and migrates into the nucleus to control transcription. Because they competitively and specifically inhibit JAK activity induced by cytokine stimulation in cells, JAK inhibitors can theoretically inhibit multiple cytokines simultaneously.

In Japan, the following types of JAK inhibitors have been approved for use: tofacitinib, baricitinib and peficitinib. Baricitinib has the added function of preventing the invasion of viruses into lung cells and immediately inhibits JAK activity induced by cytokine stimulation. Therefore, baricitinib could prevent both the invasion of viruses into the lung cells of patients infected with SARS-CoV-2 and the pathogenic induction of cytokine storms. On the other hand, tofacitinib, another JAK inhibitor, does not prevent viruses from entering the cells. The development of new JAK inhibitor drugs is currently progressing and clinical trials of these new drugs for patients with COVID-19 are underway in China [51,52].

### 8.5. Melatonin

Melatonin is secreted from the pineal body, tunes circadian rhythms through MT1/MT2 receptors, and is generally known as a sleep hormone. It also plays a role as a powerful antioxidant in cells all over the body, and it is known for its anti-inflammatory effects. Melatonin, the product of mitochondria in each cell, may prevent the intracellular invasion of CoV, by indirectly regulating the expression of ACE2, which are receptors of SARS-CoV and SARS-CoV-2. It inhibits the binding of calmodulin to ACE2 receptors and inhibits the excretion of viral external domains, which is an important process of CoV infection [53].

Melatonin also protects DNA through mitochondrial antioxidants [58,59], and it may also be useful for the recovery of mitochondria that have malfunctioned against CRS stress in COVID-19. However, melatonin is currently not approved as a therapeutic drug in Japan, and thus, future studies are needed to explore this.

### 8.6. Methylprednisolone Pulse Therapy

The systemic administration of corticosteroids is used to stop excessive inflammatory responses. It has occupied a central role in the treatment of SARS-CoV as an immunomodulatory drug.

In cases where it was administered at the appropriate time, anti-inflammatory effects, improvements on imaging, and improved oxygenation were achieved. On the other hand, premature administration of systemic corticosteroids encouraged viral growth and worsened clinical conditions. Therefore, it is important to start systemic corticosteroid therapy at the right time by taking into account the timing, type, amount and duration of administration.

Methylprednisolone pulse therapy may be one of the choices for the treatment of cytokine storms in COVlD-19 when patients are diagnosed as being in a severe state.

In rheumatic diseases, such as systemic lupus erythematosus, vasculitis syndrome and systemic scleroderma, methylprednisolone pulse therapy is usually administered to patients with an acute exacerbation of chronic inflammatory conditions, such as when presenting with cytokine storm pathology MAS, and with accelerated altered immune responses that produce auto-antibodies and immune-complex formations. These systemic pulses are also used for patients with acute circulatory failure (shock), rapidly progressing central nervous system disorders and interstitial pneumonia.

In COVID-19, clinical trials have begun in China on patients with severe ARDS [54]. However, the methylprednisolone doses of these trials are characteristically lower, between 1–2 mg/kg/day (60–120 mg/60 kg body weight/day) for 3 days. In contrast, the regular doses of methylprednisolone for pulse therapy in Japan are higher (500–1000 mg/60 kg body weight/day for three days).

### 8.7. Extracorporeal Treatments (Plasma Exchange Therapy and Continuous Hemodiafiltration)

Plasma exchange therapy is an extracorporeal treatment that is extremely effective for removing excessive proinflammatory cytokines and preventing the development of excessive inflammatory processes. In patients with both sepsis and MAS, the progression from coagulation-fibrinolysis system disorders and systemic cytolysis to DIC then multiple organ failure could be prevented. Thus, plasma exchange therapy may be useful in cytokine storm conditions due to COVID-19 [60,61].

Another effective treatment method is continuous hemodiafiltration. COVID-19 patients complicated with serious renal diseases had their lives saved by being treated with continuous hemodiafiltration [62]. This method is also known as transient renal replacement therapy, and it can adjust for uraemia and electrolyte imbalance abnormalities in acute renal failure found in patients with sepsis. It is quite useful for managing bodily fluids and electrolytes’ balance by continuous transfusion [63]. However, transfusion management failure (i.e., an excessive transfusion volume) can be directly linked to death in such patients.

## 9. Other Possible Factors for Inflammation

To maintain homeostasis, there are two stages of immune systems that act as biological defence mechanisms against bacterial and viral infections: the innate immune or inflammatory system, and the acquired immune system. During the COVID-19 pandemic, it has become apparent that there are factors that continuously induce excessive and fatal cytokinemia and chemokinemia in the innate immune response. Further investigations are needed to determine the functions of these factors, and it is possible that these induced factors exaggerate existing inflammation, thus creating a vicious cycle of inflammatory responses. We would like to stress that some of them are due to mitochondrial dysfunction and endogenous DAMPs, including cell debris and mitochondrial fracturing products such as mitochondrial DNA. In addition, the autonomic nervous system and endocrine system, such as the hypothalamic-pituitary-adrenal axis, may also be involved in the aberrant inflammatory responses since all bodily systems ordinarily respond against severe external stressors. Therefore, it is time for the global medical community to construct more flexible and comprehensive strategies to respond to the COVID-19 pandemic and effectively treat the aforementioned conditions.

The innate immune system recognises external stress factors (infection factors such as viruses and bacteria) as PAMPs (PAMPs: pathogen-associated molecular patterns), and inner stress factors (damaged cells and dead cell debris; mitochondrial fracturing products; etc.) as DAMPs (damage-associated molecular patterns) through TLRs and NLRP(NOD-like receptors)-3 inflammasome complex activation. As for the DNA of viruses and bacteria, they are recognised through the cGAS-STING (cyclic GMP-AMP synthase (cGAS) STING) pathway, while viral RNA is recognised through the RLR (Retinoic Acid-Inducible Gene-Like Receptors) pathway. As a result, proinflammatory cytokines and interferons are produced by activating transcription factors such as NF (nuclear factor) κB, AP1 (activator protein 1), and IRF (IFN regulatory factor)-3/7 with intracellular transmission mechanisms. (ASC: apoptosis-associated speck-like protein containing a CARD; pDC: plasmacytoid dendritic cells; MΦ: macrophage)

DAMPS (damage-associated molecular patterns), which are mitochondrial fracturing products, activate a number of innate immune pathways. Mitochondrial DNA (mtDNA) is released into the cytosol due to the dysfunction and destruction of mitochondria. As a result, the intracellular transmission system via cGAS (cyclic GMP-AMP synthase) and STING is activated, leading to inflammatory gene transcription activation. The mtDNA coming from phagocytised cells causes cytokines’ production via endosomal TLR-9. This results in the further production of IL-lβ, IL-18 through activation of NLRP (NOD-like receptors)-3 inflammasome [28]. (IRF: IFN regulatory factor; MyD: Myeloid differentiation primary response)

## Figures and Tables

**Table 1:** Drug therapy for COVlD-19.

Drug Therapy	Reference Number Related to the Treatment of Covid-19	Mechanism of Action
Treatment of pathogens with antiviral drugs for SARS-CoV-2		
Favipiravir (Avigan^®^®, Toyama Chemical Co., Ltd., Toyama, Japan)	[37]	
Nafamostat (Futhan^®^, Nichi-Iko Pharmaceutical Co., Ltd.,Toyama, Japan)	[38]	Prevent the binding of COVID-19 to ACE2 receptors
Cessation of cytokine storms		
Tocilizumab (Actemra^®^, Chugai Pharmaceutical Co., Ltd., To-kyo, Japan)	[25,39,40,41,42,43,44]	anti-IL-6 therapy
Sarilumab (Kevzara^®^, Sanofi S.A., Paris, France)	[44,45,46]	anti-IL-6 therapy
Anakinra (Kineret^®^, Swedish Orphan Biovitrum AB, Stockholm, Sweden)	[47]	anti-IL-1 Therapy
Canakinumab (Ilaris^®^, Novartis International AG, Basel, Switzerland)	[48]	anti-IL-1 Therapy
Adalimumab (Humira^®^, AbbVie Inc. Lake County, IL, USA)	[49,50]	anti-TNF Therapy
Infliximab (Remicade^®^, Johnson & Johnson, New Brunswick, NJ, USA)		anti-TNF Therapy
Baricitinib (Olumiant^®^, Eli Lilly and Company, Indianapolis, IN, USA)	[51,52]	JAK Inhibitor
Melatonin	[53]	May prevent intracellular invasion of CoV, by indirectly regulating the expression of ACE2
Methylprednisolone Pulse Therapy	[54]	Suppress excessive inflammatory responses

TNF: Tumor necrosis factor; JAK: Janus kinase.
